# Scanning Behavior in the Medicinal Leech *Hirudo verbana*

**DOI:** 10.1371/journal.pone.0086120

**Published:** 2014-01-21

**Authors:** Cynthia M. Harley, Daniel A. Wagenaar

**Affiliations:** 1 Department of Entomology, University of Minnesota, St Paul, Minnesota, United States of America; 2 Department of Biological Sciences, University of Cincinnati, Cincinnati, Ohio, United States of America; Claremont Colleges, United States of America

## Abstract

While moving through their environment, medicinal leeches stop periodically and wave their head or body back and forth. This activity has been previously described as two separate behaviors: one called ‘head movement’ and another called ‘body waving’. Here, we report that these behaviors exist on a continuum, and provide a detailed description of what we now call ‘scanning’. Scanning-related behavior has been thought to be involved in orientation; its function has never before been assessed. While previous studies suggested an involvement of scanning in social behavior, or sucker placement, our behavioral studies indicate that scanning is involved in orienting the leech towards prey stimuli. When such stimuli are present, scanning behavior is used to re-orient the leech in the direction of a prey-like stimulus. Scanning, however, occurs whether or not prey is present, but in the presence of prey-like stimuli scanning becomes localized to the stimulus origin. Most likely, this behavior helps the leech to gain a more detailed picture of its prey target. The display of scanning, regardless of the presence or absence of prey stimuli, is suggestive of a behavior that is part of an internally driven motor program, which is not released by the presence of sensory stimuli. The data herein include first steps to understanding the neural mechanisms underlying this important behavior.

## Introduction

Scanning movements are exhibited by many animals. In insects, for example, a leg or antenna will move in repeated circular motions in attempts to locate obstacles, food, or a way to cross a gap [1]–[4]. In predatory birds and salamanders, this type of movement occurs in the head, which is waved back and forth in efforts to find prey [5]–[8]. Similarly, many prey animals scan their environment for movement indicative of an approaching predator [9], [10], while other animals scan to localize potential mates [11]. Such behaviors are not solely visually mediated; some animals utilize tactile sensors such as limbs, whiskers, or antennae to scan their environment [2]–[4], [12]–[15]. In animals such as the hoverfly, mantis, and some hymenoptera, which cannot move their eyes, the body or head moves to scan the environment [16]–[18]. And, in humans, we scan our environment using movements of our eyes and head.

In humans, it is thought that there are two stages of visual processing. In the first stage, a limited set of basic features is processed in parallel within the whole visual field, but in limited detail. During the next stage, a smaller area is examined in greater detail [19]. The latter behavior, referred to here as ‘examining’, requires much more neural processing and occurs slowly (>300 ms in duration) [19]–[21]. In contrast, the first stage, which we call ‘scanning’, has the benefit of making it easy to spot variation within a homogeneous background and works quite fast (<30 ms in duration in some animals)[21]. As such, scanning enables objects of interest in the environment to be located quickly. These objects are then fixated upon and subsequently examined in greater detail. In humans, ‘scanning’ often describes the path that the eyes take between visual fixations (for example, see [22]. While these paths can be random, they eventually become focused on items of interest.

Here, we describe a behavior in the medicinal leech that we call ‘scanning’. During this behavior, the leech waves its body from side to side in a somewhat cyclical pattern. While this behavior has been mentioned in the leech literature as ‘searching’, ‘head waving’, ‘exploratory movements’ and ‘probing’, its true function has not been assessed, but rather assumed to be the leech looking for a suitable place to put its front sucker during locomotion [23]–[31]. We hypothesize that this behavior is equivalent to the environmental scanning behaviors mentioned above. Here, our goal was to describe scanning behavior and to examine its role in prey localization in the leech.

## Materials and Methods

Methods used within this paper have been fully described previously in Harley et al. 2011 and Harley et al. 2013 and thus, will be described briefly here.

### Animals and care

Adult Medicinal leeches (*Hirudo verbana*) were obtained from Niagara Medicinal Leeches (Niagara, NY) and maintained according to methods described by Harley et al. (2011).

### Behavioral arena

Behavioral testing was performed in a plastic saucer-shaped arena (Super Saucer, Paris co., South Paris, ME) that had a diameter of 90 cm, which was filled with water (18 +/− 0.5°C) to a depth of 20 mm (and a resultant water diameter of 75 cm). The saucer shape of this arena was chosen as it minimized the reflection of water waves. Approximately 0.2 kg of white aquarium gravel was placed on the floor of the arena as it was found that gravel aided in quiescence (Harley, *unpublished observation*). The arena was placed on an air table to isolate it from external vibrations.

### Stimuli

A stimulator used to create water waves was constructed by using a function generator (Pasco Scientific, Roseville CA drive a speaker (Pasco Scientific, Roseville CA) which contacted the water via a clear plastic circular foot (4.7 cm in diameter) which was attached to an aluminum rod. This foot was placed such that it lay flat on the surface of the water when at rest. This stimulator created waves in the testing arena containing the leech [32].

Using a piece of cardboard measuring 7 cm×7 cm we projected a shadow of approximately that same size into the arena. This encouraged the leeches to reach a quiescent state (Harley, *unpublished observations*; [33]–[35]). This state was indicated by the leech remaining in the shaded region for one minute. Once this time had passed, the shadow was removed and the stimulus was started. Individuals were given 20 minutes to reach a quiescent state and complete a trial. If a trial was not completed, due to a failure of the leech to reach quiescence within 20 minutes of introducing the individual to the arena or if the individual left the arena 3 times, it was removed from a given day's testing.

### Data Acquisition

Videos were acquired at a rate of 25 frames/sec and a resolution of 1600x1200 (2MP) using a Logitech pro 9000 webcam (Fremont, CA, USA) suspended above the arena.

### Stimulus paradigms

#### Multimodal

The arena was illuminated using a halogen flood lamp containing one 500 Watt bulb and one 250 watt bulb (McMaster-Carr, Santa Fe, CA) mounted at 1.12 meters above the arena. The wave generator was placed in the behavioral arena, 7+/−1 cm in from the edge of the water. This resulted in a multimodal stimulus which was both visual and mechanosensory. Most of the experiments presented herein are responses to an 8 Hz multimodal stimulus as we found it most effective in inducing localization behavior [36].

#### Mechanical

Like in the multimodal condition, the wave generator made waves directly in the behavioral arena, but the only light present was from four infrared (IR) LED lamps (PN850, Pinecomputer, Covina CA) placed around the arena. Additionally, we placed blackout curtain around the arena to block stray light. To further ensure the absence of any visual cues, only dim red light (<5 lux) (which leeches have a very low sensitivity to (Kretz et al., 1976)) was used in the experimental room (outside of the arena curtain) during these experiments. Our previous experiments have shown that leeches are unable to localize purely visual stimuli when they are only illuminated by infrared light [36]. Combining the low sensitivity of leeches to red lighting, and their apparent lack of ability to sense infrared lighting [36], [37], mechanical cues could reach the leech in this condition, but visual cues could not.

#### Visual

Under this condition an arena with a clear plastic bottom was mounted above the arena containing the leech. Waves were created in the top clear bottomed arena using the speaker. By lighting the clear arena with a Halogen lamp we were able to transmit the visual cues from the waves in the clear arena to the arena containing the leech without making mechanical cues available to the leech. Thus the two arenas were mechanically isolated from each other and the leech only received visual cues. In a previous study we performed an additional control whereby this assay was run in the absence of light. No orientation to the stimulus was found when light was absent from this paradigm [36].

Sample sizes for the location and duration of scanning bouts are as written in table 1.

**Table 1:** Sample sizes for different experimental variants.

	No Stimulus	2 Hz Stimulus	8 Hz Stimulus	12 Hz Stimulus
**Multimodal**	18	13	18	11
**Visual Only**	14	17	17	10
**Mechanical Only**	15	15	13	16

### Behavior Illustration

**Figure 1:**
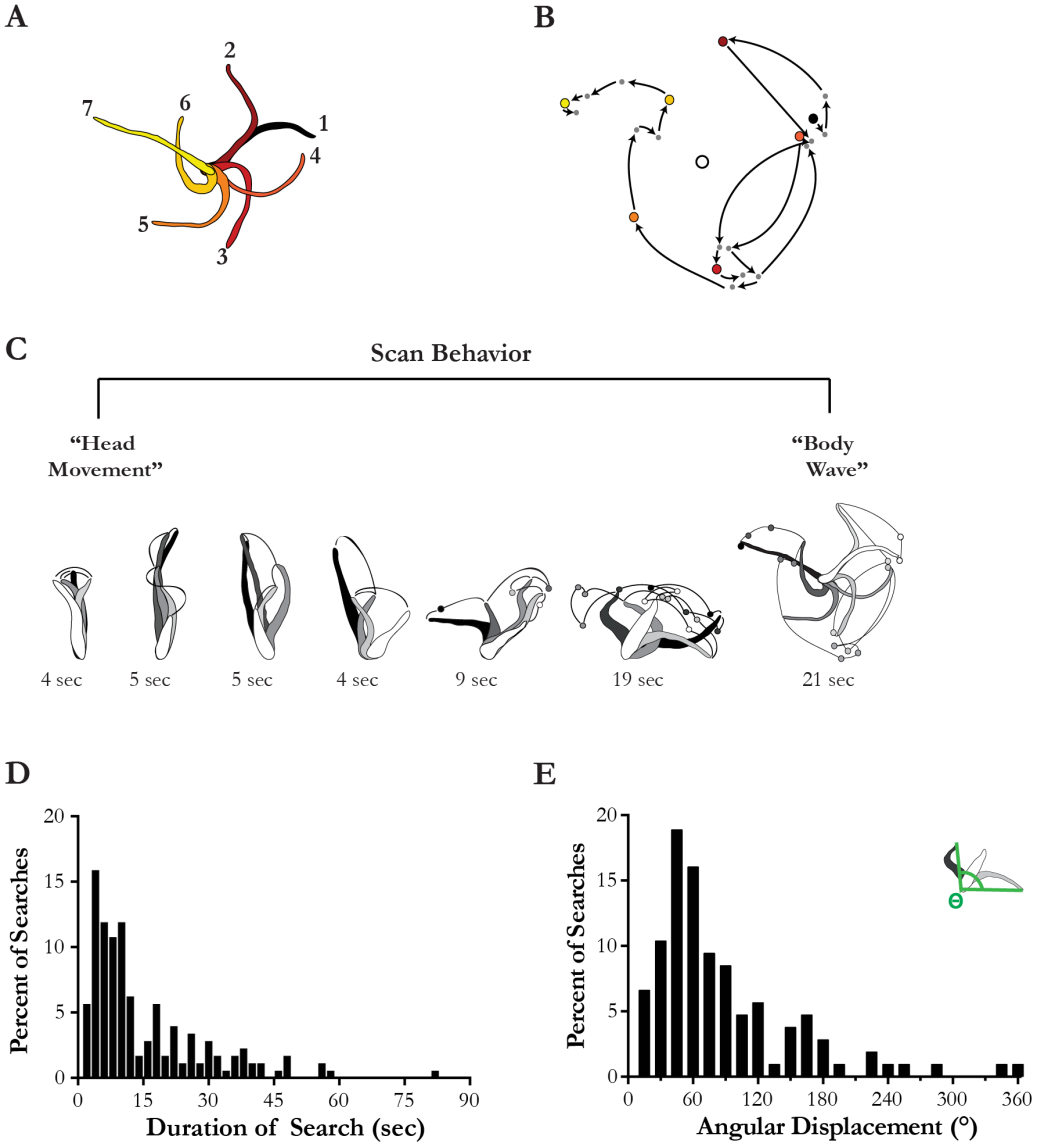
Qualitative description of scan behavior. A) Tracings of body position during every 3 seconds of a scanning behavior sequence. B) The head position during a scan sequence is marked with a circle for each second. The large colored circles correspond to positions marked in the drawing in A, the small dots represent each second in between. The open circle in the middle represents the position of the caudal sucker. Arrows denote the approximate path of the leech's head during this sequence. C) “Head movement” and “body waving” were previously described as different behaviors, but represented here on a continuum. Different sequences of scanning behavior from the same video of the same animal are shown. Each second is denoted with either a tracing of the leech's body or, when that tracing would obscure the movements of the animal, a dot. The order of these positions is denoted by the color, which starts with white and gets progressively darker until the last position shown in black. To further assess whether or not these behaviors exist on a continuum, we measured two parameters duration and angular displacement. D) The distribution of scan durations is represented here for 99 scans from 18 individuals presented with a stimulus (black). E) The distribution of angular displacements (see inset) for 99 scans from 18 individuals presented with an 8 Hz stimulus.

During scanning behavior the anterior portion of the leech's body waves back and forth while the caudal sucker remains attached to the substrate. To illustrate this movement, we traced the leech's body position every 3 seconds during a typical bout of scanning behavior (figure 1a). To further illustrate the cyclic nature of these movements, we tracked the trajectory of the leech's head during this behavior (figure 1b).

Previous studies have stated that what we call scanning can be divided into two behaviors ‘head movement’ and ‘body wave’ [27]. Here we do not separate these behaviors, because while short ‘head movements’ seem relatively easy to categorize, more vigorous ‘head movements’ could be mild ‘body waves’. This makes separating the behaviors quite difficult, as such we combine them into a single behavior called ‘scanning’. Here, we suggest that the ‘head movements’ and ‘body waves’ exist on a continuum of different extremes of scanning behavior (figure 1c). In this figure we traced the position of the leech or marked its head position at each second during the scanning bout. All illustrated scan bouts are from the same trial to minimize variability from using a different animal. Dots are used to mark head positions in trials where a tracing of the body would obscure the visual representation.

### Heading during scanning behavior

**Figure 2:**
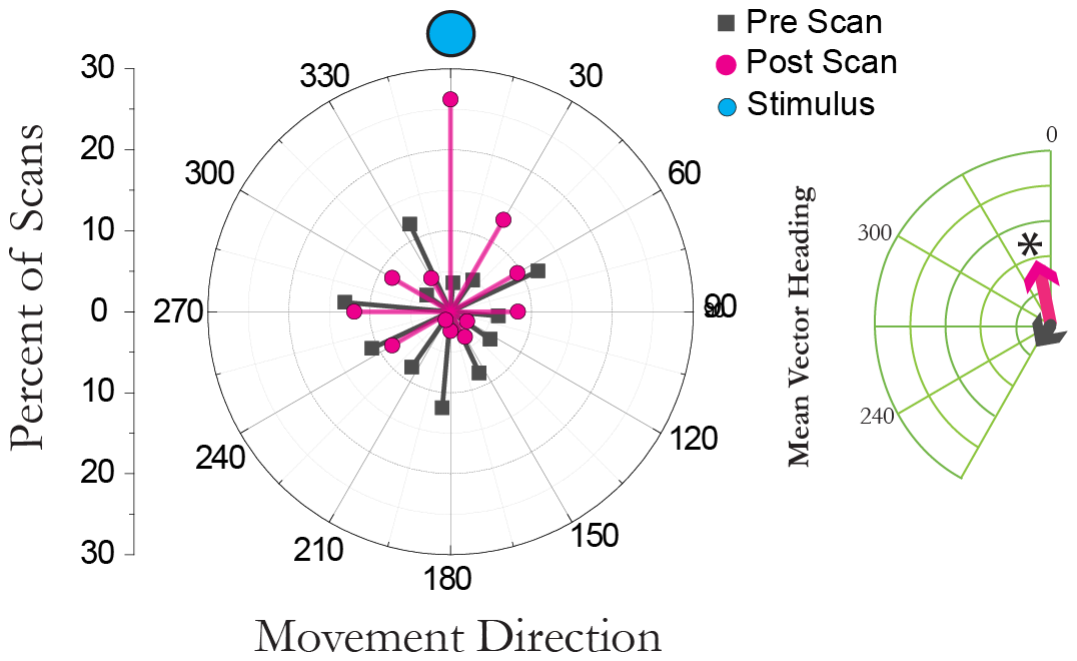
Heading changes after a sequence of scanning behavior. The leech's heading pre-scan (gray) and post scan (pink) relative to the stimulus (blue-filled circle). These data represent 99 scans from 18 animals. Gray and pink arrows represent the mean vector for the start and end of scanning bouts respectively. * indicates p<0.001 based on a Raleigh test.

The leech's heading was determined by placing a circle which had been divided into 24 sectors and printed on acetate over a video of the arena such that each sector represented a 15 degree angle (figure 2). The circle was rotated such that the stimulus was always located in sector 1. The center of the divided circle was placed over the leech's middle. The sector containing the leech's head was recorded before and after each scan bout and then translated back into an angle. This process was carried out for 18 animals and a total of 99 scans in animals which had been exposed to a continuous 8 Hz multimodal stimulus. Rayleigh tests were used to determine if distributions of angles significantly differed from random, and V-tests were used to test if heading angles were significantly directed toward the stimulus.

### Scan Duration and Location

Per methods previously described in Harley et al. 2013, the arena was divided into a grid of 32 squares. The time an individual spent scanning was determined and its location recorded. The number of seconds spent in each sector was totaled and divided by the total time that the population of animals spent searching. This measure gave the percent time spent scanning in a region. Significance was calculated by adding the mean sector value to a corrected standard deviation, but first the standard deviation was corrected by multiplying it by a corrected Z-score. Normally, the Z score would indicate how many standard deviations a value is above or below the mean and this distance is directly related to the p value. However under these conditions, because we had 32 sectors, we had to divide the desired p value by 32 and then convert that into an adjusted Z-score. The z-score for this new corrected significance level was determined to be 2.78, 2.99, 3.45 for a p of 0.1, 0.05, and 0.01 respectively. Through multiplying this value by the standard deviation and adding that to the mean, we calculated a value which set boundaries for significance. If the percent of time spent scanning exceeded these values it was determined to be significant. Scan duration was assessed only for individuals exposed to an 8 Hz stimulus (18 individuals, 99 scans) and those exposed to no stimulus (18 individuals, 84 scans).

### Angular Displacement

To examine the maximum angular displacement during scanning we recorded the angle between three points during each scan bout. These points were the caudal sucker and the furthest lateral position of the leech's head to both the clockwise and counter clockwise extremes (see figure 1e inset). These data were collected for a total of 99 scans from 18 individuals while stimulated with a 8 Hz multimodal wave stimulus using the program Tracker [38].

## Results

During periods of behavioral observation, we noted times when a given leech would cease forward motion, attach its caudal sucker to the arena floor and wave its body back and forth (figure 1a, b). Some of these body movements only involved the head while others would involve the whole body moving in a cyclical motion. In figure 1, we illustrate this behavior to create a standard description. Each second of the leech's position was either traced or, for more vigorous scans, a dot was used to mark its head position to avoid obscuring the visual representation of scanning behavior (figure 1 a–c). While the extremes of this behavioral spectrum have been separated into ‘head movement’ and ‘body waving’ in the literature, the intermediate movements are not readily separable—a more exaggerated head movement could be considered to be a weaker body wave, or a strong head movement (figure 1c). In the attempt to ascertain whether or not these behaviors were separate or on a continuum, we analyzed their duration and angular displacement. While we would expect two different behaviors to yield a bimodal distribution using these criteria, we saw no such distribution. Instead we saw a unimodal distribution indicative that these two behaviors are indeed two elements along a continuum (figures 1d, 1e). For this reason, we grouped this continuum of behaviors into a singular behavior that we call ‘scanning’.

Although cyclical sweeping-like movements are often characteristic of an orientation behavior, whether or not scanning behavior in the leech was, indeed, an orientation behavior needed further examination. Thus the leech's heading relative to a fixed stimulus source was followed before and after a scan bout. The stimulus presented was a constant 8 Hz wave stimulus (see Methods). This stimulus would be sensed by both the leech's visual and mechanosensory systems. We examined 99 scan bouts from 18 animals. Prior to bouts of scanning the leech's heading was random (a Rayleigh test yielded non-significance, P>0.3, S = 2.27; mean direction was 210° with a vector length of 0.116; shown in figure 2 grey lines). After bouts of scanning, however, the leech's heading was significantly directed towards the stimulus (Raleigh test S = 24.03, p<0.001; mean direction was 348° with a vector length of 0.37, figure 2 pink lines). The heading direction before and at the end of these bouts was significantly different (circular correlation Z = −2.66, p<0.01), strongly supporting the idea that scanning behavior plays a role in orienting the leech towards the stimulus.

**Figure 3:**
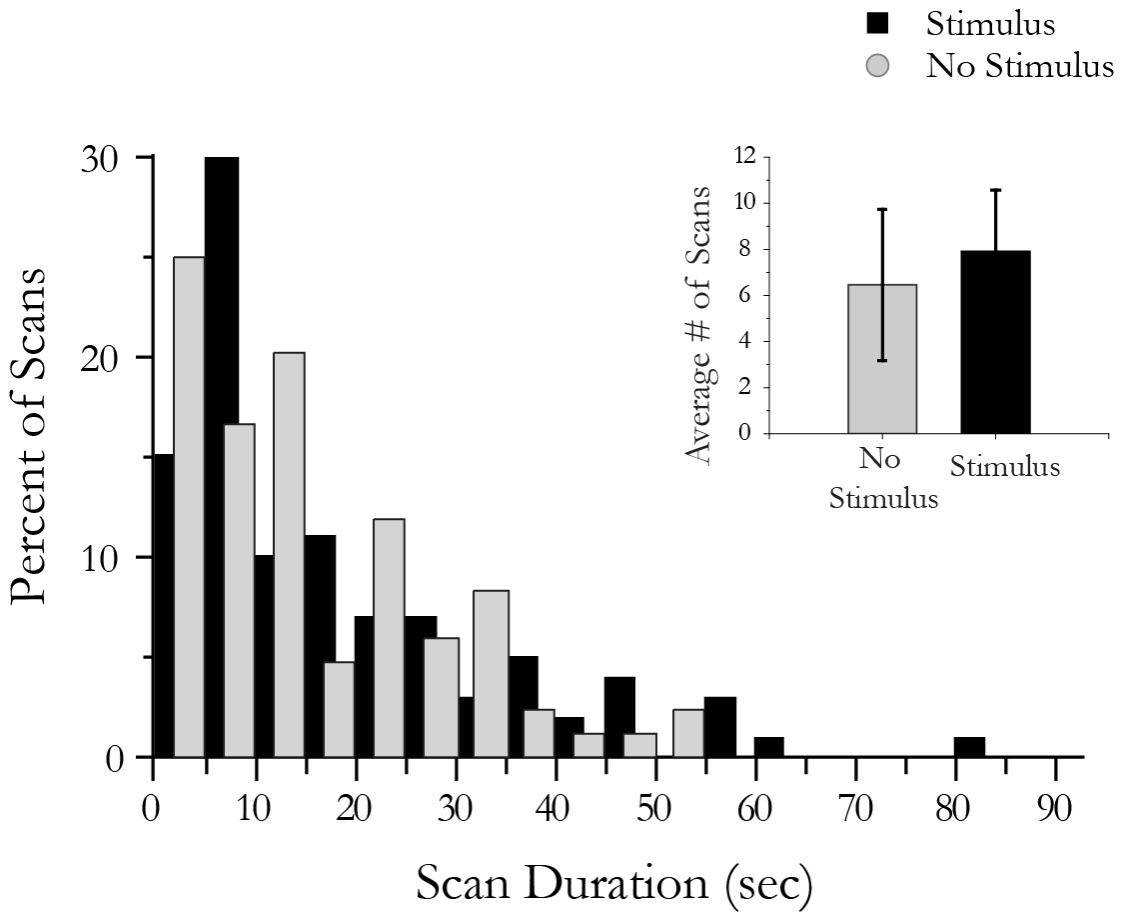
Scanning behavior occurs with the same frequency and for the same duration regardless of the presence or absence of a stimulus. The duration of scans is represented here for 99 scans from 18 individuals presented with a stimulus (black) and 84 scans in 18 individuals not presented with a stimulus (gray). We found no significant difference in scan duration when a stimulus was present (p>0.7, Kolmogrov-Smirnov test). Inset: the average number of scans for a 5 minute trial in leeches with and without a vibratory (multimodal) stimulus. We found no significant increase in the number of searches when a stimulus was present (p>0.74, Kolmogrov-Smirnov).

**Figure 4:**
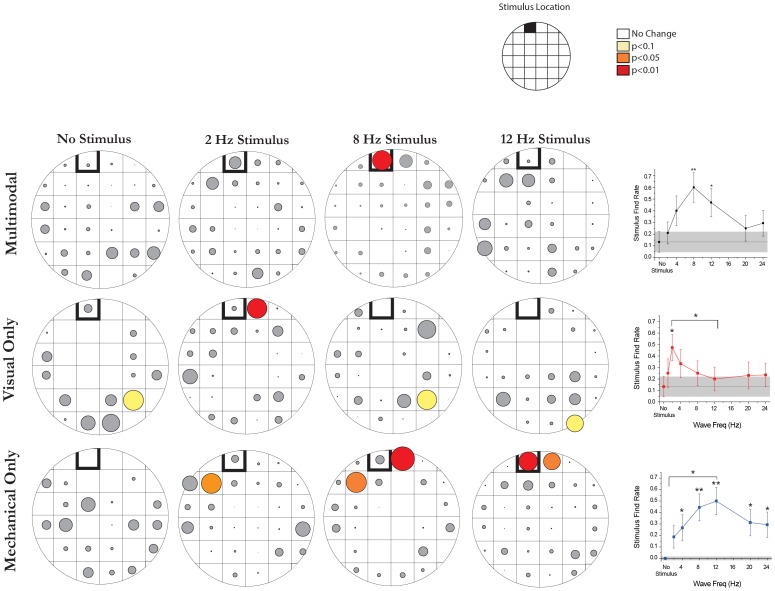
Scanning behavior becomes localized to a given stimulus. A grid of 32 squares was placed over the test arena. The amount of time a leech spent searching within each square of the grid was recorded. Because the first location of scanning behavior was influenced by the start location, we removed it from the calculation. The sum of time spent scanning by all leeches in each grid was calculated and then divided by the total time spent scanning for the group. This number resulted in the percent of scan time for a given region. Significant sectors are color coded: yellow for p<0.1; orange for p<0.05; red for p<0.01. This process was repeated for each of the following conditions: multimodal stimuli; no stimulus (n = 18), 2 Hz (n = 13), 8 Hz (n = 18), 12 Hz (n = 11); visual stimuli; no stimulus (n = 14), 2 Hz (n = 17), 8 Hz (n = 17), 12 Hz (n = 10); mechanical stimuli; no stimulus (n = 15), 2 Hz (n = 15), 8 Hz (n = 13), 12 Hz (n = 16). Line graphs represent the percent of trials resulting in finding the stimulus under each of these conditions and are reproduced from Harley 2011.

While scanning behavior appears to direct the leech towards prey-like stimuli, it is noteworthy that the behavior itself occurs regardless of whether or not prey-like stimuli were present. For example, figure 3 demonstrates that there is no significant increase in the duration or number (inset) of scan bouts when stimuli are present (black) *vs* when they are absent (grey) (for number, Kolmogrov-Smirnov, D = 0.27, p>0.73, D = 0.09, p>0.7, for number and duration, respectively). Although the number and duration of these scans did not change when the animal was presented with a stimulus (figure 3), we did observe that the location of these events was influenced by the stimulus (figure 4).

To illustrate how the location of scanning was influenced by the stimulus presented, we divided the testing arena into 32 squares and examined what percentage of the leech's time, during each trial, was spent scanning in any given region. We then took the average time spent in each quadrant for all individuals experiencing a given condition: multimodal, visual, or mechanical stimuli of varying frequencies. The size of the circle in each square represents the percent of time spent scanning in that region (fig. 4). When no stimuli were present, scans were not localized to the stimulus region (square with a thick black outline) (fig. 4 leftmost column; p>0.1, z-test for all stimulus variants, see methods for statistics). However, when stimuli were presented, the leech's scan bouts became localized to the stimulus region, but only did so under certain conditions. Scan bouts only occurred close to the stimulus source for stimuli of certain frequencies and these varied by which modality was stimulated. Searching occurred near the stimulus origin if it was a multimodal stimulus of 8 Hz (p<0.01, Z-test), a visual stimulus of 2 Hz (p<0.01 Z-test), or for any of the mechanical stimuli (p<0.01, Z test for locations marked in red, p<0.05 for locations marked in orange). This spatial display of scanning behavior was observed at the stimulus origin (p<0.01, z-test for each variant). While these results may seem highly variable, these specific stimulus conditions all have one thing in common, they are the stimuli ‘found’ most often under each of these conditions (Figure 4, far right column, Harley 2011).Thus scanning behavior, regardless of the modality used to sense the stimulus, is localized to the stimulus origin only if it is a stimulus that would ordinarily be readily localized by the leech.

## Discussion

Scanning movements are exhibited by many animals. In insects, for example, a leg or antenna will move in repeated circular motions in attempts to locate obstacles, food, or a way to cross a gap [1]–[4]. In predatory birds and salamanders, this type of movement occurs in the head, which is waved back and forth in efforts to find prey [5], [6]. And, in humans, we scan our environment using movements of our eyes and head. Here we discuss what we suggest is a similar scanning behavior in leeches (figure 1).

A previous study of leeches divided this behavior into two categories: one called “head movement” and a second called ‘body waving’ [27]. During ‘head movement’, the caudal sucker remains attached while the leech's head moves back and forth within the environment. In the leech species *Mooreobdella microstoma*, this movement occurs after each crawling step [33]. In turn, ‘body waving’ is said to occur when the leech attaches its caudal sucker to the substrate and then waves its entire body back and forth (figure 1)[27]. A previous study suggested that ‘head movement’ replaces ‘body waving’ as leeches reach adulthood [27]. We have found, however, that both behaviors are present in adult *Hirudo verbana*. Here, we attempted to separate these behaviors using duration and angular displacement as criteria (see figured 1d, 1e), however neither of these criteria provided reliable separation. Thus, we suggest that these behaviors may be different intensities of a single behavior that we have defined here as ‘scanning’ (figure 1c, d, e). Previous studies have suggested that this behavior may be involved in everything from anterior sucker placement to social behavior [27], [39], however, determination of this function has been elusive due to the stochastic nature of the behavior itself (see figure 3 and [29], [31]). Here we found that this complex behavior does become spatially restricted to the stimulus source (figure 4), however, under these conditions, it only does so for highly salient prey-like stimuli. The localization of this behavior to the source of the stimulus, suggests that scanning behavior is relevant to prey orientation. Furthermore, this scanning localization is limited to prey-like stimuli that are readily detected, suggesting that scanning only occurs in the vicinity of salient stimuli and is otherwise non-localized. Similarly, scanning bouts in other animals have been shown to become more localized when an item of interest is found during a broader search [1], [2], [13]. The benefits of localizing scanning in a small region, one that likely contains prey, would facilitate that item being examined in greater detail.

While this behavior could be considered ‘searching’ and has been previously referred to as ‘exploratory behavior’ we have opted to describe the behavior more accurately as ‘scanning’. What is the difference between the terms searching and scanning? Visual searches in humans are often divided between detection, classification and identification (for example, see [40]. Scanning often refers to the path of visual fixations that occur when viewing a scene, and thus, detection [41]. These movements occur regardless of whether or not one is ‘looking’ for something and likely served to help one find prey and avoid predators. Similarly, in the leech, scanning functions to detect possible items of interest. It happens regardless of whether or not a prey-like stimulus is present (figure 3). While it may seem that scanning behavior, in the absence of sensory stimuli, is energetically inefficient, statistical modeling of various behaviors across animal species has found that randomly occurring scans are advantageous in situations in which there is a certain level of uncertainty in the behavior of the target [42]. Thus this behavior, in leeches, would be used to survey a large environment to detect a potential food source or a moving predator. Under such conditions, it would make sense that the leech would use scan bouts to orient its subsequent movements toward sources of water disturbance when they are present (figure 2).

Scanning is not the only orientation behavior that occurs in leeches. A previous study determined that leeches orient toward stimuli during crawling bouts [32]. While at first glance two orientation behaviors may seem redundant, it is possible that they have fundamentally different but cooperative functions. One possibility is that crawling is a long-distance tracking behavior that functions to bring the scans closer to the source of the stimulus. This configuration would enable the leech both to scan the environment for relevant stimuli as well as to focus these scans within a small region. Scanning behavior could then contribute to greater detection accuracy when in close proximity or may provide additional details from near the stimulus source. In this manner, scanning and the prey orientation that occurs during crawling have different but cooperative functions.

Regardless of its function, this behavior can become activated even in the absence of sensory stimuli; thus occurring as an independent internally driven motor program. Furthermore, it is able to interrupt and modify crawling behavior (Harley, personal observation). Thus the neural control of scanning seems to have some interplay with that of crawling as it modifies crawling movements. Previous work has shown that neural commands for crawling originate in the cephalic neurons [43]–[46]. When stimulated in a semi-intact preparation, one such cephalic neuron, known as R3b2, interrupts the crawling motor pattern and causes the whole body to move in a cyclical fashion; movements similar to those associated with scanning, a behavior which also results in the cessation of forward locomotion (figure 1, Mesce et al., 2008). Furthermore, these movements become more intense with greater stimulation of this cell [44]; a mechanism which may explain the modulation in scan intensity (see figure 1). Although the function of the R3b2 has not yet been fully established, it interacts with the crawling command neuron R3b1 and can modify the activity in R3b1, and thus crawling behavior [32], [44]. The involvement of R3b2 in scanning behavior remains to be tested, however it seems to be an excellent place to initiate investigation into the neural control of scanning behavior. This is the first of many steps in the process to understand the mechanism behind this complex behavior and its interplay with other locomotor behaviors as well as the acquisition of sensory information.
